# Energy Optimization in Dual-RIS UAV-Aided MEC-Enabled Internet of Vehicles

**DOI:** 10.3390/s21134392

**Published:** 2021-06-27

**Authors:** Emmanouel T. Michailidis, Nikolaos I. Miridakis, Angelos Michalas, Emmanouil Skondras, Dimitrios J. Vergados

**Affiliations:** 1 Department of Electrical and Electronics Engineering, University of West Attica, Ancient Olive Grove Campus, 250 Thivon & P. Ralli Str, 12241 Egaleo, Greece; 2 Department of Informatics and Computer Engineering, University of West Attica, Egaleo Park Campus, Ag. Spyridonos Str, 12243 Egaleo, Greece; nikozm@uniwa.gr; 3 Department of Electrical and Computer Engineering, University of Western Macedonia, Karamanli & Ligeris, 50131 Kozani, Greece; amichalas@uowm.gr; 4 Department of Informatics, University of Piraeus, 80 Karaoli & Dimitriou St., 18534 Piraeus, Greece; skondras@unipi.gr; 5 Department of Informatics, University of Western Macedonia, Fourka Area, 52100 Kastoria, Greece; dvergados@uowm.gr

**Keywords:** computation offloading, energy efficiency, Internet of Vehicles (IoV), mobile edge computing (MEC), reconfigurable intelligent surface (RIS), unmanned aerial vehicle (UAV)

## Abstract

Mobile edge computing (MEC) represents an enabling technology for prospective Internet of Vehicles (IoV) networks. However, the complex vehicular propagation environment may hinder computation offloading. To this end, this paper proposes a novel computation offloading framework for IoV and presents an unmanned aerial vehicle (UAV)-aided network architecture. It is considered that the connected vehicles in a IoV ecosystem should fully offload latency-critical computation-intensive tasks to road side units (RSUs) that integrate MEC functionalities. In this regard, a UAV is deployed to serve as an aerial RSU (ARSU) and also operate as an aerial relay to offload part of the tasks to a ground RSU (GRSU). In order to further enhance the end-to-end communication during data offloading, the proposed architecture relies on reconfigurable intelligent surface (RIS) units consisting of arrays of reflecting elements. In particular, a dual-RIS configuration is presented, where each RIS unit serves its nearby network nodes. Since perfect phase estimation or high-precision configuration of the reflection phases is impractical in highly mobile IoV environments, data offloading via RIS units with phase errors is considered. As the efficient energy management of resource-constrained electric vehicles and battery-enabled RSUs is of outmost importance, this paper proposes an optimization approach that intends to minimize the weighted total energy consumption (WTEC) of the vehicles and ARSU subject to transmit power constraints, timeslot scheduling, and task allocation. Extensive numerical calculations are carried out to verify the efficacy of the optimized dual-RIS-assisted wireless transmission.

## 1. Introduction

In the forthcoming Internet of Vehicles (IoV) era, where multiple automobile terminals are interconnected, innovative applications will emerge, including autonomous driving, ultra-high-quality video streaming, and augmented reality (AR) [1]. As new workloads and real-time service requirements usually pose strict requirements with respect to latency, local intra-vehicle computing often struggles for timely execution of computation-intensive tasks. Moreover, a significant amount of energy is consumed that diminishes the driving range of electric vehicles [2]. To handle these challenging issues, data offloading to mobile edge computing (MEC) servers has been previously suggested [3]. In this respect, road side units (RSUs) along roads and in the vicinity of the vehicles can expedite the provision of MEC services [4].

### 1.1. Background

In recent years, there have been various contributions in the MEC-enabled IoV ecosystem. In [5], a stochastic optimization model for vehicular networks was proposed to maximize the lower bound of the expected reliability during computation offloading. In addition, a game-theoretic method was leveraged in [6] to optimize the offloading decisions in scenarios with cooperation of cloud computing and MEC. In [7], the energy consumption of RSUs in a MEC-enabled IoV was minimized using a heuristic algorithm. Moreover, an architecture for vehicular ad-hoc networks (VANETs) was introduced in [8], capable of accomplishing efficient allocation of computing resources in real-time and avoiding computation overhead. In order to jointly optimize the computing offloading and resource allocation in vehicular edge computing (VEC) network, where the vehicles act as MEC servers, a deep reinforcement learning (DRL) approach was presented in [9]. The processing delay in software-defined networking (SDN)-based and fiber-wireless (FiWi)-enabled VEC networks was minimized in [10] through a load-balancing task of-floading scheme. By trading on hybrid vehicle-to-infrastructure (V2I) and vehicle-to-vehicle (V2V) connections along with geolocation information, a computation offloading protocol was also constructed in [11] for reliable data retrieval in VEC scenarios. Furthermore, an edge intelligence-enabled IoV was described in [12] and an online algorithm based on Lyapunov optimization was proposed to decrease the total network delay.

While fruitful results have been described in previous work on MEC in IoV, the highly dynamic network topologies of IoV drastically influence the data offloading process. More importantly, the communication links between vehicles and ground RSUs (GRSUs) can be blocked in propagation environments with large obstacles and dispersed nodes. In this direction, hovering aerial RSUs (ARSUs) relying on unmanned aerial vehicles (UAVs) have great potential to attain a higher chance of line-of-sight (LoS) communication across different terrains thus sufficiently extending the radio coverage between vehicles and MEC servers [13,14,15]. In previous work, a UAV was employed to assist an access point (AP) in providing MEC services to ground users (GUs) in an energy-efficient manner [16]. Identical MEC schemes were also envisioned to optimize the energy consumption [17], the maximum delay [18], the task completion time [19], the average latency [20], and the computation efficiency [21]. Furthermore, energy harvesting and wireless power transfer (WPT) were introduced to prolong the network’s operation time and an optimization problem was formulated to maximize the computation rate [22]. Moreover, an Internet of Things (IoT) scheme was studied in [23], where UAVs were employed to collect data from IoT devices and forward these data to multiple distributed MEC-based APs. In addition, an energy-optimized IoT scenario was presented in [24], where a UAV integrated an edge server and provided computation services to ground IoT nodes. A social IoV (SIoV) network was proposed in [25] and jointly optimized the resource allocation and the UAV’s trajectory. In addition, an SDN-based offloading strategy for vehicular networks was presented in [26] and the task execution time was minimized subject to quality of service (QoS) and energy consumption constraints. Beyond the conventional orthogonal multiple access (OMA) scenarios, a non-orthogonal multiple access (NOMA) setup was investigated in [27], whereas the stochastic offloading concept extended the deterministic binary and partial task offloading in [28]. Furthermore, the benefits of massive multiple-input–multiple-output (MIMO) transmission were underlined in [29]. Nevertheless, challenges remain to be addressed to ensure highly reliable communication links, especially in uncontrollable vehicular environments and urban areas, where scattering objects (e.g., buildings, poles, trees, hills or human bodies) may induce severe signal attenuation or signal blockage.

On the other hand, with the rapid evolution of radio frequency (RF) micro electro-mechanical systems (MEMs), the programmable and reconfigurable meta-surfaces have emerged, among which the reconfigurable intelligent surface (RIS) technology has recently received unprecedented attention [30,31]. More specifically, RIS stands for a thin metasurface consisting of a large number of passive and phase-controllable reflecting elements, each of which can be digitally controlled to reflect the incident signals with adjusted phase shifts and thus reconfigure the wireless propagation environment in favor of signal transmission. Contrary to conventional active relaying, RIS leverages passive reflection and leads to cost-effective, low-complexity, and energy-efficient implementations with high array gain and low noise. Previously, RIS-assisted UAV communication systems were envisioned [32,33,34], without accentuating MEC applications. In the context of MEC networks, the adoption of RIS units in various wireless network setups with propagation-induced impairments has also been investigated. A single-cell scenario with multiple single-antenna devices and a multi-antenna AP was considered in [35] and an optimization problem was formulated to minimize latency, under practical constraints related with the total edge computing capability and the phase shift design. In [36], an IoT MEC system with multiple devices was investigated capable of supporting passive beamforming via a RIS unit in the computational offloading stage. In this system, the sum computational bits were maximized in a partial computational offloading manner. An RIS-aided MEC-enabled flexible time-sharing scheme that enables both NOMA and time-division multiple-access (TDMA) transmission via data division was proposed in [37] and the sum delay of the users was minimized under discrete-phase constraints of the RIS. Additionally, a RIS-assisted MEC system that can handle learning-driven tasks was presented in [38] and involved a multi-antenna intelligent edge server and multiple single-antenna users affiliated with machine learning (ML) tasks. In this system, the learning error was minimized by taking into account the transmit power constraints and the phase shifts of the RIS. Nevertheless, the aforementioned works are unsuitable for UAV-based networks and cannot capture the air-to-ground channel features. As the UAVs fly in a three-dimensional (3-D) space and above rooftops, especial geometrical and mobility characteristics are introduced. Table 1 provides a brief description of the key elements of the aforementioned previous works, which give emphasis either on MEC IoV architectures, where the vehicles’ computing tasks are completed without cooperation of UAVs [5,6,7,8,9,10,11,12]; or UAV-enabled MEC network architectures that do not include RIS units [16,17,18,19,20,21,22,23,24,25,26,27,28,29]; or RIS-assisted UAV networks without MEC capabilities [32,33,34]; or RIS-assisted MEC network architectures with only ground-based nodes [35,36,37,38].

### 1.2. Contribution

To the best of the authors’ knowledge, the area of computation offloading for RIS-based UAV-aided IoV is unexplored. As newer network architectures are indispensable, this paper proposes an IoV framework, which includes a UAV-based ARSU and relies on RIS units. This framework may find practical application in beyond fifth generation (B5G) mission-critical scenarios, where multiple resource-limited vehicles should execute computationally intensive tasks by using the processing capabilities of MEC servers. As the communication links between vehicles and GRSUs may be blocked, an ARSU can bring flexibility, additional computing resources, and extended radio coverage. RIS units are also employed, in order to further enhance the connectivity and reliability by reshaping the propagation environment in favor of signal transmission. More specifically, the main contributions of this paper can be summarized as follows:

- A novel dual-MEC IoV architecture is proposed, where a rapidly deployed and dynamically repositioned UAV-based ARSU equipped with a MEC server facilitates the computation offloading and also acts as an intermediate decode-and-forward (DF) aerial relay enabling the communication between vehicles and a GRSU. Full offloading is applied and a trade-off between energy consumption and delay is obtained by efficiently using the computing resources at both ARSU and GRSU.
- In practice, the direct communication links between vehicles (ARSU) and ARSU (GRSU) may be vulnerable to fading and blockage effects due to large objects in the propagation environment. Thus, the proposed architecture leverages a dual-RIS deployment strategy to assist the direct communication. It is considered that one RIS unit is positioned close to the vehicles and a second RIS unit is positioned towards GRSU. Owing to the dynamic and highly mobile vehicular environment, imperfect estimation of the reflection phases is introduced. Hence, wireless transmission via the RIS units with phase errors is assumed. In order to obtain a 3-D realistic geometrical positioning of the vehicles, ARSU, GRSU, and RIS units, while accurately modeling the mobility characteristics, velocity and distance vectors are utilized.
- Moreover, this paper formulates a multi-variable optimization problem to minimize the weighted total energy consumption (WTEC) from both the vehicles and ARSU perspective and elongate their endurance. In this respect, the Lagrange dual method along with a subgradient-based algorithm are leveraged to provide optimal solutions for the transmit power allocation, timeslot scheduling, and task allocation. Moreover, an asymptotic analysis of the WTEC is included as the number of reflecting elements increases. The numerical results illustrate the total computation-based and communication-based delay (TCCD) and WTEC, focus on the benefits of the dual-RIS-based data offloading, and affirm the efficiency of the optimization method.

### 1.3. Structure

The rest of this paper is organized as follows. In Section 2, the system model is introduced, the geometrical characteristics and the mobility model are outlined, and the computation offloading model is presented. Section 3 describes the wireless transmission model. In Section 4, the optimization problem is formulated. Numerical results are provided in Section 5. Finally, conclusions and future research directions are drawn in Section 6.

## 2. System Model

In this paper, a MEC-enabled IoV is considered consisting of *K* vehicles moving along a unidirectional road segment, where the distribution of the vehicles follows a Poisson distribution [39]. The latency-critical computation task of the *k*-th vehicle with 1≤k≤K can be only executed remotely within the maximum allowable latency (task deadline) $\eta_{k}$ by performing task offloading to MEC servers. Thus, a fixed grid-powered GRSU with powerful computation capacity is situated along the road. In the proposed IoV scenario, high attenuation obstructs the direct link between the vehicles and GRSU. Hence, a flying ARSU with certain energy and computing limitations is also employed to facilitate the provision of MEC services and also enable vehicle-to-GRSU networking via relaying. More specifically, the ARSU initially designates the portion of vehicles’ offloaded tasks that can timely execute with its own computing resources and then forwards the remaining part of these tasks to GRSU. In this direction, the ARSU uses an adequately large data buffer, in order to separately store the offloaded and processed data. As LoS air-to-ground propagation cannot be continuously ensured in urban and dense-urban environments, two RIS units, one unit close to the vehicles and a second unit close to the GRSU, are also installed on surrounding building walls to assist the direct communication between the *k*-th vehicle (ARSU) and ARSU (GRSU).

### 2.1. Geometrical Characteristics and Mobility Model

**Figure 1:**
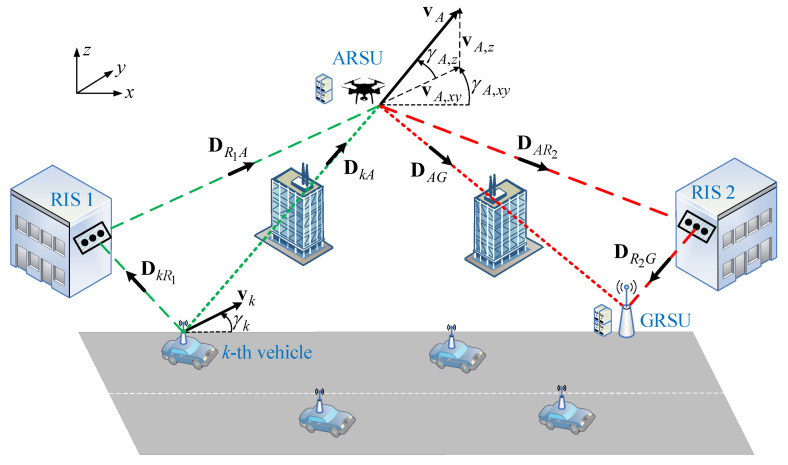
A simple representation of the dual-RIS UAV-aided MEC-enabled IoV architecture.

The 3-D geometrical features of the proposed IoV architecture are depicted in Figure 1, where the (x,y,z) axes define the coordinate system. To aid our analysis, the subscripts *k*, *A*, $R_{1}$, *G*, and $R_{2}$ with 1≤k≤K are associated with the *k*-th vehicle, ARSU, 1st RIS, GRSU, and 2nd RIS, respectively. It is considered that $\left(x_{k}\left[n\right],y_{k}\left[n\right],0\right)$, $\left(x_{A}\left[n\right],y_{A}\left[n\right],z_{A}\left[n\right]\right)$, $\left(x_{R_{1}},y_{R_{1}},z_{R_{1}}\right)$, $\left(x_{G},y_{G},z_{G}\right)$ and $\left(x_{R_{2}},y_{R_{2}},z_{R_{2}}\right)$ are the coordinates of the *k*-th vehicle, ARSU, 1st RIS, GRSU, and 2nd RIS, respectively. Let $D_{ab}$ denote the distance vector between two arbitrary points *a* and *b*. Then, $∥D_{kA}\left[n\right]∥=\sqrt{{\left(x_{k}\left[n\right]-x_{A}\left[n\right]\right)}^{2}+{\left(y_{k}\left[n\right]-y_{A}\left[n\right]\right)}^{2}+z_{A}^{2}\left[n\right]}$ is the distance between the *k*-th vehicle and ARSU and $∥\cdot ∥$ is the Euclidean norm. Note that the distances $∥D_{kR_{1}}\left[n\right]∥$, $∥D_{R_{1}A}\left[n\right]∥$, $∥D_{AG}\left[n\right]∥$, $∥D_{AR_{2}}\left[n\right]∥$ and $∥D_{R_{2}G}\left[n\right]∥$ can be defined accordingly. It is assumed that the *k*-th vehicle and ARSU are moving with velocities $\upsilon_{k}$ and $\upsilon_{A^{\prime }}$, respectively, in the direction in the azimuth domain determined by the angles $\gamma_{k}$ and $\gamma_{A,xy^{\prime }}$, respectively. Additionally, the hovering, diving, and rising operations of ARSU can described by the elevation angle $\gamma_{A,z}$. Then, $v_{k}=\upsilon_{k}{\left[cos\gamma_{k},sin\gamma_{k},0\right]}^{T}$ and $v_{A}=\upsilon_{A}\left[cos\gamma_{A,xy}cos\gamma_{A,z},sin\gamma_{A,xy}cos\gamma_{A,z},sin\gamma_{A,z}\right]$ stand for the velocity vectors of the *k*-th vehicle and ARSU, respectively. In practice, the ARSU should not notably draw away from its initial position, since its movement may affect the connectivity with the vehicles and GRSU. It is assumed that the initial locations of the vehicles are known to the ARSU for designing its trajectory, whereas the fixed locations of the 1st RIS and 2nd RIS are known as well. For convenience, we use a sufficiently small constant τ to divide the ARSU’s flying period *T* into *N* timeslots. (In this paper, the case that $\eta_{k}=T$ is only considered ∀k). During each timeslot, it is assumed that both the *k*-th vehicle and the ARSU are static. The coordinates of the *k*-th vehicle are updated as $x_{k}\left[n+1\right]=x_{k}\left[n\right]+\upsilon_{k}cos\gamma_{k}\tau$ and $y_{k}\left[n+1\right]=y_{k}\left[n\right]+\upsilon_{k}sin\gamma_{k}\tau$, where n∈{1,2,…N}. Furthermore, the coordinates of the ARSU are updated as $x_{A}\left[n+1\right]=x_{A}\left[n\right]+\upsilon_{A}cos\gamma_{A,xy}cos\gamma_{A,z}\tau$, $y_{A}\left[n+1\right]=y_{A}\left[n\right]+\upsilon_{A}sin\gamma_{A,xy}cos\gamma_{A,z}\tau$ and $z_{A}\left[n+1\right]=z_{A}\left[n\right]+\upsilon_{A}sin\gamma_{A,z}\tau$.

In this paper, a rotary-wing UAV is considered as an ARSU, owing to its higher mobility compared to that of a fixed-wing UAV. Based on [40], the propulsion energy consumption in the *n*-th timeslot can be modeled as

(1) $$\begin{array}{c} E_{fl}\left[n\right]=\tau (P_{0}\left(1+\frac{3{∥v}_{A,xy}\left[n\right]{∥}^{2}}{v_{\mathrm{tip}}^{2}}\right)+\frac{1}{2}d_{r}s\rho G{∥v}_{A,xy}\left[n\right]{∥}^{3}\\ +P_{1}\sqrt{\sqrt{1+\frac{{∥v}_{A,xy}\left[n\right]{∥}^{4}}{4v_{0}^{2}}}-\frac{{∥v}_{A,xy}\left[n\right]{∥}^{2}}{2v_{0}^{2}}}+P_{2}{∥v}_{A,z}\left[n\right]∥), \end{array}$$

where $P_{0}$ is the blade profile power, $\upsilon_{tip}$ is the tip speed of the rotor blade, $d_{r}$ is the fuse-lage drag ratio, *s* is the rotor solidity, ρ is the air density, *G* is the rotor disc area, $P_{1}$ is the induced power, $\upsilon_{0}$ is the mean rotor induced velocity, $P_{2}$ is the descending/ascending power, and $v_{A,xy[n]}$ and $v_{A,z[n]}$ are the horizontal and vertical ARSU velocity vectors, respectively, with $v_{A}\left[n\right]=v_{A,xy}\left[n\right]+v_{A,z}\left[n\right]$.

### 2.2. Computation Offloading Model

It is considered that $l_{k}$ defines a particular computation task of the *k*-th vehicle and $b_{k}$ is the task-input data size (in bits). The maximum central processing unit (CPU) frequency at ARSU is denoted as $f_{A,max}$, whereas $c_{A}>0$ is the number of required CPU cycles per bit at ARSU. As the intra-vehicle computational resources are limited, the *k*-th vehicle fully offloads to ARSU and GRSU (via relaying) its task. Thus, $\beta_{k}\left[n\right]b_{k}\left[n\right]$ and $\left(1-\beta_{k}\left[n\right]\right)b_{k}\left[n\right]$ computation bits are allocated for computing at ARSU and at GRSU, respectively, where $\beta_{k}\left[n\right]$ is the offloading task assignment ratio. It is noted that the transmission delay and the energy consumption for data downloading are omitted, since the size of the output computed data is assumed to be significantly smaller than that of the input data for computing. In addition, the computation delay at GRSU can be neglected owing to its computing capabilities, while the time required for performing task partitioning is negligible compared to the overall latency and is omitted as well.

In order to implement the computation offloading, the TDMA protocol is adopted. This protocol has been widely used in IoV networks [41] and MEC networks [16,19,25] and can obviate transmission collisions, contention-induced overhead, and interference among vehicles, while retaining short delivery delay. Thus, we divide each timeslot into *K* equal durations ${\left\{\tau_{k}\left[n\right]\right\}}_{k=1}^{K}$ with $\sum_{k=1}^{K}\tau_{k}\left[n\right]=\tau$ and $\tau_{k}\left[n\right]=\tau_{k,off}\left[n\right]+\tau_{A,off}\left[n\right]$ where $\tau_{k,off}\left[n\right]$ is the transmission time for offloading $b_{k}\left[n\right]$ from the *k*-th vehicle to ARSU and $\tau_{k,A,off}\left[n\right]$ is the transmission time for offloading $\left(1-\beta_{k}\left[n\right]\right)b_{k}\left[n\right]$ from ARSU to GRSU. It is assumed that the computation delay $\tau_{k,cA}\left[n\right]=c_{A}\beta_{k}\left[n\right]b_{k}/f_{A,max}$ at ARSU can span a duration $\tau_{k}\left[n\right]$. Then, we obtain the following inequalities:

(2) $$0\leq \left\{\tau_{k,off}\left[n\right],\tau_{k,A,off}\left[n\right],\tau_{k,cA}\left[n\right]\right\}\leq \frac{\tau }{K},$$

(3) $$\tau_{k,off}\left[n\right]+\tau_{k,A,off}\left[n\right]\leq \frac{\tau }{K}.$$

The energy consumed by the *k*-th vehicle and ARSU, respectively, for data offloading in the *n*-th timeslot can be expressed as:

(4) $$E_{k,off}\left[n\right]=p_{k,off}\left[n\right]\tau_{k,off}\left[n\right],$$

(5) $$E_{k,A,off}\left[n\right]=p_{k,A,off}\left[n\right]\tau_{k,A,off}\left[n\right],$$

where $p_{k,off}\left[n\right]$ and $p_{k,A,off}\left[n\right]$ is the transmit power of the *k*-th vehicle and ARSU, respectively, for bits offloading in the *n*-th timeslot. Moreover, the energy consumption for ARSU computing in the *n*-th timeslot can be written as [42]

(6) $$E_{k,cA}\left[n\right]=P_{k,cA}\tau_{k,cA}\left[n\right]\equiv \kappa_{A}c_{A}^{3}K^{2}{\left(\beta_{k}\left[n\right]b_{k}\left[n\right]\right)}^{3}\tau^{-2},$$

where $P_{k,cA}=\kappa_{A}f_{A,max}^{3}$ is the power consumption of the CPU at ARSU [42] and $\kappa_{A}>0$ is the chip-dependent effective capacitance coefficient that is affiliated with the ARSU.

## 3. Wireless Transmission Model

It is considered that the *k*-th vehicle, the ARSU, and the GRSU are equipped with single omni-directional antennas, whereas the RIS units employ multiple reflecting elements as well as a wireless controller for the dynamic adjustment of the phase shift of each element. Without loss of generality, it is assumed that the 1st and 2nd RIS units have the same number *L* of reflecting elements. It is also assumed that the channel gain remains unchanged in each timeslot, since the *k*-th vehicle and the ARSU shift over an insignificantly short distance. Thus, during the flying period, the wireless radio channel can be represented by a series of channel snapshots, where each snapshot is associated with a particular position of the *k*-th vehicle and ARSU.

### 3.1. Direct Links without RIS Units

In order to model the channel fading behavior in air-to-ground propagation scenarios, several statistical distributions, e.g., Rician, Rayleigh, and Nakagami-*m* distributions, have been previously used [43,44,45]. Among these distributions, the Nakagami-*m* distribution was experimentally validated in UAV-based scenarios [46], whereas it also provides flexibility in several environments by including Rayleigh distribution as a special case or by approximately describing Rician fading. Thus, this paper considers that the channel behavior of the direct link between the *k*-th vehicle (ARSU) and ARSU (GRSU) is subjected to Nakagami-*m* fading conditions. Then, the cumulative distribution function (CDF) of the instantaneous SNR received at the ARSU can be expressed as [47]

(7) $$F_{\gamma_{th},kA}\left(\gamma_{th}\right)=1-\frac{\Gamma (m_{kA},\frac{m_{kA}\gamma_{th}}{{\bar{\gamma }}_{kA}\left[n\right]})}{\Gamma (m_{kA})},$$

where $\Gamma \left(y,x\right)=\Gamma \left(y\right)exp\left(-x\right)\sum_{k=0}^{y-1}\left(x^{k}/k!\right)$ is the upper incomplete Gamma function [48], eq. (8.350/2), $\Gamma \left(\alpha \right)=\int_{0}^{\infty }t^{\alpha -1}e^{-t}dt$ is the complete Gamma function [48], eq. (8.310/1), $m_{kA}\geq 1/2$ is the Nakagami-*m* fading parameter, $\gamma_{th}=2^{r_{t}}-1$ is the SNR threshold, $r_{t}$ is the target rate (in bps/Hz), and ${\bar{\gamma }}_{kA}\left[n\right]$ is the average SNR. By adopting the Friis’s formula [49], ${\bar{\gamma }}_{kA}\left[n\right]$ can be expressed as

(8) $${\bar{\gamma }}_{kA}\left[n\right]=\frac{p_{k,off}\left[n\right]}{N_{0}}\beta_{0}{∥D_{kA}\left[n\right]∥}^{-\sigma_{kA}},$$

where $N_{0}$ is the variance of the additive white Gaussian noise (AWGN) at ARSU, $\beta_{0}$ is the channel gain at a reference distance $d_{0}=1\;m$, and $\sigma_{kA}$ is the pathloss exponent of the link between the *k*-th vehicle and ARSU. (Without loss of generality, it is assumed that $N_{0}$ is the variance of AWGN at any IoV node).

### 3.2. Indirect Links through RIS Units

The channel between a UAV and a RIS unit is mostly dominated by LoS links, since the UAV usually flies at high altitudes and the RIS unit is mounted on the facade of a building. However, multipath propagation may also exist in urban and dense-urban environments with tall buildings acting as effective scatterers. In addition, local scattering effects may influence the channels between an RIS unit and vehicles, whereas an additional LoS link can be also established [32,33]. Thus, in this paper, the channel between the *k*-th vehicle (*l*-th element of 1st RIS) and the *l*-th element of 1st RIS (ARSU) is modeled as a Rician fading channel accounting for both the LoS and non-line-of-sight (NLoS) components. Each RIS element should be designed to maximize the reflection strength and steer the reflection angle towards the ARSU (or GRSU). However, it is assumed that the phase shifts induced by the channels cannot be perfectly evaluated and/or the desired phases cannot be precisely set. Quantization phase errors are considered and thus only a discrete set of $2^{q}$ phases is configured with q≥1, where *q* is the number of quantization bits. It is assumed that the phase error $\Theta_{l}$ is uniformly distributed over $\left[-2^{-q}\pi ,2^{-q}\pi \right]$. Then, as shown in [50], the composite channel between the *k*-th vehicle and ARSU via the 1st RIS is equivalent to a direct channel with Nakagami scalar fading and can be expressed as

(9) $$h_{kR_{1}A}\left[n\right]\overset{\Delta }{\;=}\frac{1}{L}\sum_{l=0}^{L-1}|h_{kl}\left[n\right]||h_{lA}\left[n\right]|exp\left(j\Theta_{l}\right),$$

where $h_{kl}\left[n\right]\left(h_{lA}\left[n\right]\right)$ is the channel gain in the *n*-th timeslot for the link between the *k*-th vehicle (*l*-th element of the 1st RIS) and the *l*-th element of the 1st RIS (ARSU). It is assumed that the phase errors ${\left\{\Theta_{l}\right\}}_{l=1}^{L}$ are independent and identically distributed (i.i.d.) with a common characteristic function expressed as a sequence of complex numbers ${\left\{\varphi_{\zeta }\right\}}_{\zeta \in Z}$, which are referred to as trigonometric (or circular) moments [51], with $|\varphi_{\zeta }|\leq 1\;$∀ζ∈Z. The size of the RIS units is assumed to be small relative to the propagation links. Thus, the reflector elements experience identical large-scale fading. Let $m_{kR_{1}A}$ and ${\bar{\gamma }}_{kR_{1}A}\left[n\right]$ denote the Nakagami-*m* fading parameter and average SNR, respectively, of the composite channel. Then, the CDF of the instantaneous SNR received at ARSU is expressed as [47]

(10) $$F_{\gamma_{th},kR_{1}A}\left(\gamma_{th}\right)=1-\frac{\Gamma \left(m_{kR_{1}A},\frac{m_{kR_{1}A}}{{\bar{\gamma }}_{kR_{1}A}\left[n\right]}\gamma_{th}\right)}{\Gamma \left(m_{kR_{1}A}\right)},$$

where

(11) $$m_{kR_{1}A}\overset{\Delta }{=}\frac{L}{2}\frac{\theta_{1}^{2}\alpha_{kR_{1}}^{2}\alpha_{R_{1}A}^{2}}{1+\theta_{2}-2\theta_{1}^{2}\alpha_{kR_{1}}^{2}\alpha_{R_{1}A}^{2}},$$

(12) $${\bar{\gamma }}_{kR_{1}A}\left[n\right]\overset{\Delta }{\;=}\frac{p_{k,off}\left[n\right]}{N_{0}}L^{2}\theta_{1}^{2}E[|h_{kl}{\left[n\right]|]}^{2}E[|h_{lA}\left[n\right]{\left|\right]}^{2},$$

(13) $$a_{kR_{1}}=\sqrt{\frac{\pi }{4(K_{kR_{1}}+1)}}{}_{1}F_{1}\left(-\frac{1}{2},1;K_{kR_{1}}\right),$$

(14) $$a_{R_{1}A}=\sqrt{\frac{\pi }{4(K_{R_{1}A}+1)}}{}_{1}F_{1}\left(-\frac{1}{2},1;K_{R_{1}A}\right),$$

(15) $$E[|h_{kl}\left[n\right]\left|\right]=a_{kR_{1}}\sqrt{\beta_{0}{∥D_{kR_{1}}\left[n\right]∥}^{-\sigma_{kR_{1}}}},$$

(16) $$E[|h_{lA}\left[n\right]\left|\right]=a_{R_{1}A}\sqrt{\beta_{0}{∥D_{kR_{1}}\left[n\right]∥}^{-\sigma_{R_{1}A}}},$$

$\theta_{1}=\frac{sin(2^{-q}\pi )}{2^{-q}\pi }$, $\theta_{2}=\frac{sin(2^{-q+1}\pi )}{2^{-q+1}\pi }$, E[·] is the expectation operator, $\sigma_{kR_{1}}\left(\sigma_{R_{1}A}\right)$ is the path-loss exponent of the link between the *k*-th vehicle (1st RIS) and 1st RIS (ARSU), ${}_{1}F_{1}\left(.,.;.\right)$ is the Kummer confluent hypergeometric function [52], eq. (2.18), and $K_{kR_{1}}\left(K_{R_{1}A}\right)$ is the Rician factor for the link between the *k*-th vehicle (1st RIS) and 1st RIS (ARSU). Using (7) and (10), the CDF of the instantaneous SNR received at ARSU accounting for both the direct link as well as the link via the 1st RIS can be expressed as follows [53], eq. (33)

(17) $$\begin{array}{c} F_{\gamma_{th},kA,kR_{1}A}\left(\gamma_{th}\right)=\frac{{\left(\frac{m_{kA}}{{\bar{\gamma }}_{kA}\left[n\right]}\right)}^{m_{kA}}{\left(\frac{m_{kR_{1}A}}{{\bar{\gamma }}_{kR_{1}A}\left[n\right]}\right)}^{m_{kR_{1}A}}\gamma_{th}^{m_{kA}+m_{kR_{1}A}}}{\Gamma \left(m_{kA}+m_{kR_{1}A}+1\right)}\\ \times \Phi_{2}\left(m_{kA},m_{kR_{1}A};m_{kA}+m_{kR_{1}A}+1;-\frac{m_{kA}\gamma_{th}}{{\bar{\gamma }}_{kA}\left[n\right]};-\frac{m_{kR_{1}A}\gamma_{th}}{{\bar{\gamma }}_{kR_{1}A}\left[n\right]}\right), \end{array}$$

where $\Phi_{2}\left(\cdot ;\cdot ;\cdot ;\cdot \right)$ is the Humbert confluent hypergeometric series $\Phi_{2}$ and can be easily computed using the procedure in [54]. Using (12), the effective rate (in bps/Hz) can be obtained as follows

(18) $$r_{kA,kR_{1}A}\left[n\right]=r_{t}\left[1-F_{\gamma_{th},kA,kR_{1}A}\left(\gamma_{th}\right)\right].$$

Using (18) and properly replacing the indices, the effective rate $r_{AG,AR_{2}G}\left[n\right]$ accounting for both the direct link between ARSU and GRSU as well as the link via the 2nd RIS can be similarly defined. It is assumed that the number of bits that are offloaded to ARSU (GRSU) does not exceed the offloading rate capabilities of the corresponding channel. Thus, it follows that:

(19) $$b_{k}\left[n\right]\leq \tau_{k,off}\left[n\right]r_{kA,kR_{1}A}\left(p_{k,off}\left[n\right]\right),$$

(20) $$\left(1-\beta_{k}\left[n\right]\right)b_{k}\left[n\right]\leq \tau_{k,A,off}\left[n\right]r_{AG,AR_{2}G}\left(p_{k,A,off}\left[n\right]\right).$$

It is considered that the vehicles control their transmit power according to a signal-to-noise-ratio (SNR) threshold, in order to maintain an acceptable QoS. In this paper, maximal-ratio combining (MRC) is adopted. Thus, the requirement ${\bar{\gamma }}_{kA}\left[n\right]+{\bar{\gamma }}_{kR_{1}A}\left[n\right]\geq \gamma_{kA}$ should be satisfied [55]. Using the aforementioned inequality, as well as (8) and (11)–(16), we obtain

(21) $$p_{k,off}\left[n\right]\geq \frac{1}{P_{1}\left[n\right]+P_{2}\left[n\right]},$$

where

(22) $$P_{1}\left[n\right]=\frac{\beta_{0}{∥D_{kA}\left[n\right]∥}^{-\sigma_{kA}}}{\left(2^{r_{t}}-1\right)N_{0}},$$

(23) $$P_{2}\left[n\right]={\left(\frac{L\theta_{1}\beta_{0}\pi }{4}\right)}^{2}\frac{{∥D_{kR_{1}}\left[n\right]∥}^{-\sigma_{kR_{1}}}{∥D_{R_{1}A}\left[n\right]∥}^{-\sigma_{R_{1}A}}}{\left(2^{r_{t}}-1\right)\left(K_{kR_{1}}+1\right)\left(K_{R_{1}A}+1\right)N_{0}}{\left[{}_{1}F_{1}\left(-\frac{1}{2},1;K_{kR_{1}}\right){}_{1}F_{1}\left(-\frac{1}{2},1;K_{R_{1}A}\right)\right]}^{2}.$$

From (21), one concludes that $p_{k,off}^{min}\left[n\right]=1/\left(P_{1}\left[n\right]+P_{2}\left[n\right]\right)$ is the lower bound of the transmit power of the *k*-th vehicle for an acceptable QoS in the *n*-th timeslot.

### 3.3. Asymptotic Rate

In order to support a massive number of vehicles in future MEC-enabled IoV scenarios, a vast number of discrete reflecting elements at each RIS unit is required. Thus, this paper derives the asymptotic rate, as *L* increases. As L→∞ we obtain [56], eq. (1.7)

(24) $$\begin{array}{c} F_{\gamma_{th},kA,kR_{1}A}^{asymp}\left(\gamma_{th}\right)=\lim_{L\to \infty }F_{\gamma_{kA,t}}\left(\gamma_{th}\right)≃\frac{{\left(\frac{m_{kA}}{{\bar{\gamma }}_{kA}\left[n\right]}\right)}^{m_{kA}}{\left(\frac{m_{kR_{1}A}}{{\bar{\gamma }}_{kR_{1}A}\left[n\right]}\right)}^{m_{kR_{1}A}}\gamma_{th}^{m_{kA}+m_{kR_{1}A}}}{\Gamma \left(m_{kA}+m_{kR_{1}A}+1\right)}\\ \times \Phi_{2}\left(m_{kA},m_{kR_{1}A};m_{kA}+m_{kR_{1}A};-\frac{m_{kA}\gamma_{th}}{{\bar{\gamma }}_{kA}\left[n\right]};-\frac{m_{kR_{1}A}\gamma_{th}}{{\bar{\gamma }}_{kR_{1}A}\left[n\right]}\right)\\ ≃\frac{{\left(\frac{m_{kA}}{{\bar{\gamma }}_{kA}\left[n\right]}\right)}^{m_{kA}}{\left(\frac{m_{kR_{1}A}}{{\bar{\gamma }}_{kR_{1}A}\left[n\right]}\right)}^{m_{kR_{1}A}}\gamma_{th}^{m_{kA}+m_{kR_{1}A}}}{\Gamma \left(m_{kA}+m_{kR_{1}A}+1\right)}\times {}_{1}F_{1}\left(m_{kA},m_{kA}+m_{kR_{1}A};-\frac{m_{kA}\gamma_{th}}{{\bar{\gamma }}_{kA}\left[n\right]}\right)\\ ≃\frac{{\left(\frac{m_{kA}}{{\bar{\gamma }}_{kA}\left[n\right]}\right)}^{m_{kA}}{\left(\frac{m_{kRA}}{{\bar{\gamma }}_{kR_{1}A}\left[n\right]}\right)}^{m_{kR_{1}A}}\gamma_{th}^{m_{kA}+m_{kR_{1}A}}}{\Gamma \left(m_{kA}+m_{kR_{1}A}+1\right)}\exp(-\frac{m_{kA}\gamma_{th}}{{\bar{\gamma }}_{kA}\left[n\right]}). \end{array}$$

Using (24), we obtain the asymptotic expression of the effective rate in (18) as

(25) $$r_{kA,kR_{1}A}^{asymp}\left[n\right]=r_{t}\left[1-F_{\gamma_{th},kA,kR_{1}A}^{asymp}\left(\gamma_{th}\right)\right].$$

## 4. Minimization of Energy Consumption

This section formulates a multi-variable optimization problem to minimize the WTEC of the proposed dual-RIS UAV-aided MEC-enabled IoV architecture. This problem is explicitly subjected to transmit power allocation, achievable rate (target offloading rate), timeslot scheduling, and task allocation.

### 4.1. Problem Formulation

The optimization problem can be formulated as:

(26a) $$\left(P1\right):\min_{P,\tau ,\beta_{k}\left[n\right]}E_{total}=\sum_{n=1}^{N}\left(\left(\sum_{k=1}^{K}w_{k}E_{k,off}\left[n\right]\right)+w_{A}E_{A}\left[n\right]\right)$$

(26b) $$s.t.\;p_{k,off}^{\min}\left[n\right]\leq p_{k,off}\left[n\right]\leq p_{k,off}^{\max}\left[n\right],$$

(26c) $$p_{k,A,off}^{\min}\left[n\right]\leq p_{k,A,off}\left[n\right]\leq p_{k,A,off}^{\max}\left[n\right],$$

(26d) $$0\leq \tau_{k,off}\left[n\right]\leq \frac{\tau }{K},$$

(26e) $$0\leq \tau_{k,A,off}\left[n\right]\leq \frac{\tau }{K},$$

(26f) $$0\leq \beta_{k}\left[n\right]\leq \frac{\tau f_{A,\max}}{Kc_{A}b_{k}\left[n\right]},$$

(26g) $$\tau_{k,off}\left[n\right]+\tau_{k,A,off}\left[n\right]\leq \frac{\tau }{K},$$

(26h) $$b_{k}\left[n\right]\leq \tau_{k,off}\left[n\right]r_{kA,kR_{1}A}\left(p_{k,off}\left[n\right]\right),$$

(26i) $$\left(1-\beta_{k}\left[n\right]\right)b_{k}\left[n\right]\leq \tau_{k,A,off}\left[n\right]r_{AG,AR_{2}G}\left(p_{k,A,off}\left[n\right]\right),$$

where $E_{total}$ is the WTEC, $P≜\left\{p_{k,off}\left[n\right],p_{k,A,off}\left[n\right]\right\},$ $\tau ≜\left\{\tau_{k,off}\left[n\right],\tau_{k,A,off}\left[n\right]\right\},$ and $\beta_{k}\left[n\right]$ are the optimizing variables, $w_{k}\geq 0$ and $w_{A}\geq 0$ constitute the weight factors of energy consumption of *k*-th vehicle and ARSU, respectively, $p_{k,off}^{\min}\left[n\right]$ can be defined using (21), $p_{k,A,off}^{\min}\left[n\right]$ is the minimum transmit power of ARSU and can be defined using (21) and properly replacing the indices, $p_{k,off}^{\max}$ and $p_{k,A,off}^{\max}$ are the maximum transmit powers of *k*-th vehicle and ARSU, respectively, and

(27) $$E_{A}\left[n\right]=\sum_{k=1}^{K}\left(E_{k,cA}\left[n\right]+E_{k,A,off}\left[n\right]\right)$$

is the total communication-related and computation-related energy consumption of ARSU in the *n*-th timeslot. The weights can be modified according to energy demands and tradeoffs of an envisioned IoV application and also provide priority/fairness among the vehicles. Thus, $w_{k}\left(w_{A}\right)$ should be increased to save more energy, as long as the *k*-th vehicle’s (ARSU’s) battery is running low. It is noted that the constraints (26b) and (26c) specify the range of transmit power of the *k*-th vehicle and ARSU, the constraints (26d), (26e), and (26g) indicate the transmission delay limitations, the constraint (26f) designates the range of the task assignment ratio, and the constraints (26h) and (26i) describe the data offloading limitations. In general, the propulsion energy consumption, which is defined in (1), is remarkable and is significantly affected by the ARSU’s trajectory, i.e., the time-variant location of the ARSU during the flight period. However, in this paper, a pre-determined ARSU’s trajectory is considered and the optimization of the trajectory, which can further decrease the WTEC, is left as a future work. Thus, the propulsion energy consumption is excluded from the optimization process.

**Lemma** **1.** *Problem (P1) is a convex problem.*

**Proof.**  From (4)–(6), (26a), and (27), it follows that the objective function of problem (P1) is convex with respect to P and $\beta_{k},$ since its Hessian matrix is positive semidefinite. Moreover, the mathematical expressions in (26b)–(26g) are linear. In addition, using (22) and (23), (17) can be written as [57], eq. (7.2) (p. 446)

(28) $$\begin{array}{ccc} F_{\gamma_{th},kA,kR_{1}A}\left(\gamma_{th}\right) & = & \frac{p_{k,off}^{-\left(m_{kA}+m_{kR_{1}A}\right)}\left[n\right]}{\Gamma \left(m_{kA}+m_{kR_{1}A}+1\right)}{\left(\frac{m_{kA}}{P_{1}\left[n\right]}\right)}^{m_{kA}}{\left(\frac{m_{kR_{1}A}}{P_{2}\left[n\right]}\right)}^{m_{kR_{1}A}}\\ & & \times \Phi_{2}\left(m_{kA},m_{kR_{1}A};m_{kA}+m_{kR_{1}A}+1;-\frac{m_{kA}}{P_{1}\left[n\right]p_{k,off}\left[n\right]};-\frac{m_{kR_{1}A}}{P_{2}\left[n\right]p_{k,off}\left[n\right]}\right)\\ & = & \frac{p_{k,off}^{-\left(m_{kA}+m_{kR_{1}A}\right)}\left[n\right]}{\Gamma \left(m_{kA}+m_{kR_{1}A}+1\right)}{\left(\frac{m_{kA}}{P_{1}\left[n\right]}\right)}^{m_{kA}}{\left(\frac{m_{kR_{1}A}}{P_{2}\left[n\right]}\right)}^{m_{kR_{1}A}}\\ & & \times \sum_{v=0}^{\infty }\sum_{u=0}^{\infty }\frac{{\left(m_{kA}\right)}_{v}{\left(m_{kR_{1}A}\right)}_{u}}{{\left(m_{kA}+m_{kR_{1}A}+1\right)}_{v+u}v!u!}{\left(-\frac{m_{kA}}{P_{1}\left[n\right]}\right)}^{v}{\left(-\frac{m_{kR_{1}A}}{P_{2}\left[n\right]}\right)}^{u}p_{k,off}^{-\left(v+u\right)}\left[n\right]\\ & = & \sum_{v=0}^{\infty }\sum_{u=0}^{\infty }\frac{{\left(\frac{m_{kA}}{P_{1}\left[n\right]}\right)}^{m_{kA}+v}{\left(\frac{m_{kR_{1}A}}{P_{2}\left[n\right]}\right)}^{m_{kR_{1}A}+u}{\left(m_{kA}\right)}_{v}{\left(m_{kR_{1}A}\right)}_{u}p_{k,off}^{-\left(m_{kA}+m_{kR_{1}A}+v+u\right)}\left[n\right]}{\Gamma \left(m_{kA}+m_{kR_{1}A}+1\right){\left(m_{kA}+m_{kR_{1}A}+1\right)}_{v+u}v!u!},\; \end{array}$$

where ${\left(x\right)}_{n}\equiv \Gamma \left(x+n\right)/\Gamma \left(x\right)$ is the Pochhammer symbol [58] (p. 256). Using (28), the second derivative of $F_{\gamma_{th},kA,kR_{1}A}\left(\gamma_{th}\right)$ with respect to $p_{k,off}\left[n\right]$ can be expressed as

 

(29) $$\begin{array}{c} \frac{\partial^{2}F_{\gamma_{th},kA,kR_{1}A}\left(\gamma_{th}\right)}{\partial p_{k,off}^{2}\left[n\right]}=\sum_{v=0}^{\infty }\sum_{u=0}^{\infty }\frac{{\left(\frac{m_{kA}}{P_{1}\left[n\right]}\right)}^{m_{kA}+v}{\left(\frac{m_{kR_{1}A}}{P_{2}\left[n\right]}\right)}^{m_{kR_{1}A}+u}{\left(m_{kA}\right)}_{v}{\left(m_{kR_{1}A}\right)}_{u}}{\Gamma \left(m_{kA}+m_{kR_{1}A}+1\right){\left(m_{kA}+m_{kR_{1}A}+1\right)}_{v+u}v!u!}\\ \times \left(m_{kA}+m_{kR_{1}A}+v+u\right)\left(m_{kA}+m_{kR_{1}A}+v+u+1\right){\left(p_{k,off}\left[n\right]\right)}^{-\left(m_{kA}+m_{kR_{1}A}+v+u+2\right)}.\; \end{array}$$

As $\partial^{2}F_{\gamma_{th},kA,kR_{1}A}\left(\gamma_{th}\right)/\partial p_{k,off}^{2}\left[n\right]>0,$$F_{\gamma_{th},kA,kR_{1}A}\left(\gamma_{th}\right)$ is a strictly convex function of $p_{k,off}\left[n\right].$ Thus, one concludes that the right-hand-side of (26h) and (26i) is concave. Consequently, Problem (P1) is a convex problem.    □

### 4.2. Problem Solution

This paper leverages the Lagrangian dual method to solve Problem (P1). First, the non-negative dual variables ${\left\{\chi_{\delta ,k,n}\right\}}_{\delta =1}^{3}$ are introduced, each associated with one of the constraints in (26g)–(26i). Then, the Lagrange function of problem (P1) can be written as $L\left(P,\tau ,\beta_{k}\left[n\right],x_{1},x_{2},x_{3}\right)=$

(30) $$\begin{array}{c} \sum_{n=1}^{N}\sum_{k=1}^{K}\left[w_{k}p_{k,off}\left[n\right]\tau_{k,off}\left[n\right]+w_{A}\left(\kappa_{A}c_{A}^{3}K^{2}{\left(\beta_{k}\left[n\right]b_{k}\left[n\right]\right)}^{3}\tau^{-2}+p_{k,A,off}\left[n\right]\tau_{k,A,off}\left[n\right]\right)\right]\\ +\sum_{n=1}^{N}\sum_{k=1}^{K}\chi_{2,k,n}b_{k}\left[n\right]+\sum_{n=1}^{N}\sum_{k=1}^{K}\chi_{3,k,n}\left(1-\beta_{k}\left[n\right]\right)b_{k}\left[n\right]-\sum_{n=1}^{N}\sum_{k=1}^{K}\chi_{1,k,n}\frac{\tau }{K}+\sum_{n=1}^{N}\sum_{k=1}^{K}\chi_{1,k,n}\tau_{k,off}\left[n\right]\\ +\sum_{n=1}^{N}\sum_{k=1}^{K}\chi_{1,k,n}\tau_{k,A,off}\left[n\right]-\sum_{n=1}^{N}\sum_{k=1}^{K}\chi_{2,k,n}\tau_{k,off}\left[n\right]r_{kA,kR_{1}A}\left(p_{k,off}\left[n\right]\right)\\ -\sum_{n=1}^{N}\sum_{k=1}^{K}\chi_{3,k,n}\tau_{k,A,off}\left[n\right]r_{AG,AR_{2}G}\left(p_{k,A,off}\left[n\right]\right), \end{array}$$

where $x_{1},$$x_{2},$ and $x_{3}$ denote the sets of $\chi_{1,k,n},$$\chi_{2,k,n},$ and $\chi_{3,k,n},$ respectively. Thus, the dual function of problem (P1) can be expressed as

(31a) $$\xi \left(x_{1},x_{2},x_{3}\right)=\min_{P,t,\beta_{k}}L\left(P,t,\beta_{k},x_{1},x_{2},x_{3}\right)$$

(31b) s.t.(26b)−(26f)

Moreover, the dual problem of problem (P1) can be written as follows

(32a) $$P1-\mathrm{dual}:\;\;\;\max_{x_{1},x_{2},x_{3}}\;\xi \left(x_{1},x_{2},x_{3}\right)$$

(32b) $$s.t.\;\left\{x_{1},x_{2},x_{3}\right\}⪰0$$

Since problem (P1) is convex, it satisfies the Slater’s condition [59]. As a strong duality between (P1) and (P1-dual) can be observed, the optimal solution of problem (P1) is obtained by solving problem (P1-dual). In addition, the dual function is obtained by solving the problem in (31a) and (31b) for arbitrary values of ${\left\{x_{\delta }\right\}}_{\delta =1}^{3}$. This particular problem can be decomposed into a set of *KN* independent subproblems and these sub-problems can be further decomposed into three subproblems as  

$\left(L1\right):\min_{\tau_{k,off}\left[n\right],p_{k,off}\left[n\right]}\left(w_{k}p_{k,off}\left[n\right]+\chi_{1,k,n}\right)\tau_{k,off}\left[n\right]$

$\;\;\;\;\;\;\;\;\;\;\;\;-\chi_{2,k,n}\tau_{k,off}\left[n\right]r_{kA,kR_{1}A}\left(p_{k,off}\left[n\right]\right)$

s.t.(26b),(26d)

$\left(L2\right):\min_{\tau_{k,A,off}\left[n\right],p_{k,A,off}\left[n\right]}\left(w_{A}p_{k,A,off}\left[n\right]+\chi_{1,k,n}\right)\tau_{k,A,off}\left[n\right]$

$\;\;\;\;\;\;\;\;\;\;\;\;\;-\chi_{3,k,n}\tau_{k,A,off}\left[n\right]r_{AG,AR_{2}G}\left(p_{k,A,off}\left[n\right]\right)$

s.t.(26c),(26e)

$\left(L3\right):\min_{b_{k,A}\left[n\right]}w_{A}\kappa_{A}c_{A}^{3}K^{2}{\left(\beta_{k}\left[n\right]b_{k}\left[n\right]\right)}^{3}\tau^{-2}+\chi_{3,k,n}\left(1-\beta_{k}\left[n\right]\right)b_{k}\left[n\right]$

s.t.(26f)  

The aforementioned subproblems are convex. Hence, the Karush–Kuhn–Tucker (KKT) conditions may be imposed on these subproblems for finding their optimal solutions. The optimal transmit power $p_{k,off}^{*}\left[n\right]$ of the *k*-th vehicle can be obtained by applying KKT conditions, setting the derivative of the Lagrangian of subproblem (L1) with respect to $p_{k,off}\left[n\right]$ to zero and applying numerical solving methods. Then, $\tau_{k,off}^{*}\left[n\right]$ can be obtained by substituting $p_{k,off}^{*}\left[n\right]$ into subproblem (L1) and is expressed as

(33) $$\tau_{k,off}^{*}\left[n\right]=\{\begin{array}{c} =\frac{\tau }{K},w_{k}p_{k,off}^{*}\left[n\right]+\chi_{1,k,n}-\chi_{2,k,n}r_{kA,kR_{1}A}\left(p_{k,off}^{*}\left[n\right]\right)<0\\ \in \left[0,\frac{\tau }{K}\right],w_{k}p_{k,off}^{*}\left[n\right]+\chi_{1,k,n}-\chi_{2,k,n}r_{kA,kR_{1}A}\left(p_{k,off}^{*}\left[n\right]\right)=0\\ =0,w_{k}p_{k,off}^{*}\left[n\right]+\chi_{1,k,n}-\chi_{2,k,n}r_{kA,kR_{1}A}\left(p_{k,off}^{*}\left[n\right]\right)>0 \end{array}.$$

Using (33), we also obtain the following optimal solution:

(34) $$E_{k,off}^{*}\left[n\right]=p_{k,off}^{*}\left[n\right]\tau_{k,off}^{*}\left[n\right].$$

By applying KKT-based conditions, the solution to the subproblem (L2) can be similarly obtained.

To derive a closed-form solution and provide insights into problem (L1), we present an asymptotic analysis as *L* increases.

**Proposition** **1.**  *The optimal transmit power of the k-th vehicle and the offloading time, when L→∞ can be, respectively, obtained as*

(35) $$p_{k,off,asymp}^{*}\left[n\right]={\left[\frac{P_{1}\left[n\right]P_{3}}{m_{kA}}\cdot W\left(\frac{m_{kA}}{P_{1}\left[n\right]P_{3}}\sqrt[{P_{3}}]{\frac{\chi_{2,k,n}P_{4}}{P_{1}^{m_{kA}}\left[n\right]P_{2}^{m_{kR_{1}A}}\left[n\right]}}\right)\right]}_{p_{k,off}^{\min}\left[n\right]}^{p_{k,off}^{\max}},$$

(36) $$\tau_{k,off,asymp}^{*}\left[n\right]=\{\begin{array}{c} =\frac{\tau }{K},w_{k}p_{k,off,asymp}^{*}\left[n\right]+\chi_{1,k,n}-\chi_{2,k,n}r_{kA,kR_{1}A}^{asymp}\left(p_{k,off,asymp}^{*}\left[n\right]\right)<0\\ \in \left[0,\frac{\tau }{K}\right],w_{k}p_{k,off,asymp}^{*}\left[n\right]+\chi_{1,k,n}-\chi_{2,k,n}r_{kA,kR_{1}A}^{asymp}\left(p_{k,off,asymp}^{*}\left[n\right]\right)=0\\ =0,w_{k}p_{k,off,asymp}^{*}\left[n\right]+\chi_{1,k,n}-\chi_{2,k,n}r_{kA,kR_{1}A}^{asymp}\left(p_{k,off,asymp}^{*}\left[n\right]\right)>0 \end{array},$$

*where*

(37) $$P_{3}=\left(3-m_{kA}-m_{kR_{1}A}\right),$$

(38) $$P_{4}=\frac{r_{t}\left(m_{kA}+m_{kR_{1}A}\right)m_{kA}^{m_{kA}}m_{kR_{1}A}^{m_{kR_{1}A}}}{w_{k}\Gamma \left(m_{kA}+m_{kR_{1}A}+1\right)},$$

*and $W\left(\cdot \right)$ is the Lambert function [60].*

**Proof.**  As L→∞, the achievable rate can be defined using (25). Using (25) instead of (18), applying KKT conditions, solving the equation $\partial L_{1}/\partial p_{k,off}\left[n\right]=0,$ and performing some mathematical manipulations, we can derive the optimal solution in (35). Then, the optimal solution in (36) is obtained by substituting $p_{k,off}^{*}\left[n\right]$ into subproblem (L1).    □

Next, we provide a recommendation for the energy-efficient offloading task assignment ratio. By solving subproblem (L3) with the aid of KKT conditions, the following optimal solution is obtained

(39) $$\beta_{k}^{*}\left[n\right]=\frac{\tau }{Kb_{k}\left[n\right]}\sqrt{\frac{\chi_{3,k,n}}{3w_{A}\kappa_{A}c_{A}^{3}}}.$$

One observes that ARSU processes fewer task-input data, as $w_{A}$ increases. In addition, the ARSU should not perform full offloading to GRSU for computing, since $\beta_{k}^{*}\left[n\right]>0.$ Thus, $\frac{\tau }{K}\sqrt{\frac{\chi_{3,k,n}}{3w_{A}\kappa_{A}c_{A}^{3}}}$ bits should be computed at ARSU for minimum WTEC.

As arbitrary dual variables are considered heretofore, the optimal dual variables can be obtained by solving problem P1-dual. Since problem P1-dual is generally non-differentiable, the iterative ellipsoid method [59] is adopted to obtain an optimal solution. It is considered that the subgradient of the objective function is represented by ${\left(\Delta x_{1}^{T},\Delta x_{2}^{T},\Delta x_{3}^{T}\right)}^{T},$ where

(40a) $$\Delta x_{1}=b_{k}\left[n\right]-\tau_{k,off}\left[n\right]r_{kA,kR_{1}A}\left(p_{k,off}\left[n\right]\right),$$

(40b) $$\Delta x_{2}=\left(1-\beta_{k}\left[n\right]\right)b_{k}\left[n\right]-\tau_{k,A,off}\left[n\right]r_{AG,AR_{2}G}\left(p_{k,A,off}\left[n\right]\right),$$

(40c) $$\Delta x_{3}=\tau_{k,off}\left[n\right]+\tau_{k,A,off}\left[n\right]-\frac{\tau }{K}.$$

Since the optimal solution $\tau^{*}$ is not unique, the following linear programming problem is formulated:

(41a) $$\left(P2\right):\min_{\tau }\sum_{n=1}^{N}\sum_{k=1}^{K}w_{k}E_{k,off}\left[n\right]+w_{A}E_{k,A,off}\left[n\right]$$

(41b) $$s.t.\;0\leq \tau_{k,off}\left[n\right]\leq \frac{\tau }{K},$$

(41c) $$0\leq \tau_{k,A,off}\left[n\right]\leq \frac{\tau }{K},$$

(41d) $$\tau_{k,off}\left[n\right]+\tau_{k,A,off}\left[n\right]\leq \frac{\tau }{K},$$

(41e) $$b_{k}\left[n\right]\leq \tau_{k,off}\left[n\right]r_{kA,kR_{1}A}\left(p_{k,off}^{*}\left[n\right]\right),$$

(41f) $$\left(1-\beta_{k}^{*}\left[n\right]\right)b_{k}\left[n\right]\leq \tau_{k,A,off}\left[n\right]r_{AG,AR_{2}G}\left(p_{k,A,off}^{*}\left[n\right]\right).$$

In order to obtain the optimal solution to principal problem (P1), problem (P2) should be solved. In this regard, the subgradient-based Algorithm 1 is proposed to derive an optimal solution. The complexity of Algorithm 1 is due to Steps 4 to 6, the complexity of which is $O\left(KN\right),$$O\left(KN\right),$ and $O\left(K^{2}N^{2}\right)$ [59], respectively. Hence, Algorithm 1 is characterized by an entire complexity of $O\left(K^{4}N^{4}\right).$ On the other hand, the complexity in Step 9 depends on solving problem (P2) by CVX [61].
**Algorithm 1:** Optimal Solution to Problem (P1).1.**Set** the values of IoV parameters and the value of the tolerant threshold ε.2.**Initialize** the iteration index, the dual variables ${\left\{x_{\delta }\right\}}_{\delta =1}^{3},$ and the ellipsoid.Then, calculate the achievable rate using (18).3.**Repeat**4.Solve subproblems (L1) and (L2) with KKT conditions and obtain $P^{*}$ and $\tau^{*}.$Then, use (39) and obtain $\beta_{k}^{*}\left[n\right].$ Calculate the WTEC.5.Solve problem P1-dual and calculate the subgradients of the objectivefunction and the constraints.6.Update ${\left\{x_{\delta }\right\}}_{\delta =1}^{3}$ based on the ellipsoid method.7.**End Repeat** until convergence.8.Let ${\left\{x_{\delta }^{*}\right\}}_{\delta =1}^{3}\leftarrow {\left\{x_{\delta }\right\}}_{\delta =1}^{3}$9.Update $P^{*}$ by solving subproblems (L1) and (L2) with KKT conditions.Use (39) and obtain $\beta_{k}^{*}\left[n\right].$Then, derive $\tau^{*}$ by solving problem (P2) by CVX and obtain the optimalWTEC.

## 5. Numerical Results and Discussion

**Figure 2:**
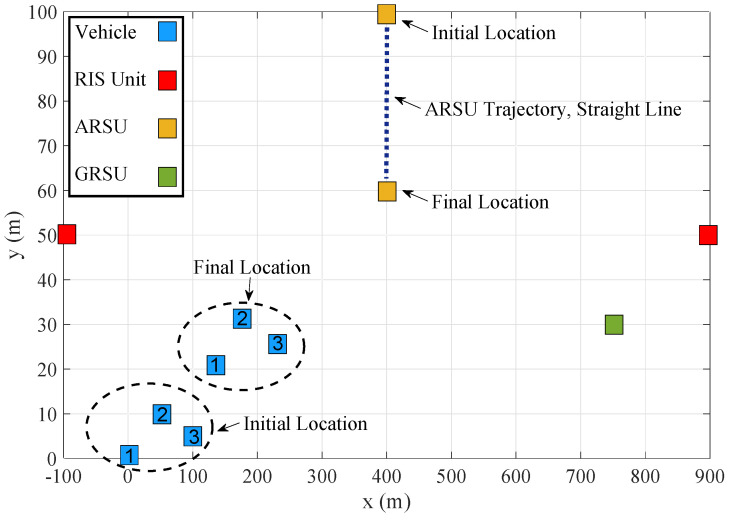
The projection of the proposed IoV architecture on the xy plane with pre-determined benchmark trajectories of three vehicles and the aerial road side unit (ARSU).

This section provides results to demonstrate the impact of the key network parameters on the TCCD $\tau_{TCCD}=\sum_{n=1}^{N}\sum_{k=1}^{K}\tau_{k}\left[n\right]$ and on the non-optimized and optimized WTEC. These results were obtained using MATLAB 2020a and the popular MATLAB-based CVX modeling framework for disciplined convex programming [61]. Figure 2 shows the simulation setup with pre-determined benchmark trajectories of three vehicles and the ARSU over the horizontal plane within a given rectangular area of 1000m×100m. The initial coordinates (in meters) of the 1st vehicle, 2nd vehicle, 3rd vehicle, ARSU, 1st RIS, GRSU, and 2nd RIS are $\left(x_{1}\left[1\right],y_{1}\left[1\right],z_{1}\left[1\right]\right)=\left(0,0,0\right),$$\left(x_{2}\left[1\right],y_{2}\left[1\right],z_{2}\left[1\right]\right)=\left(50,10,0\right),$$\left(x_{3}\left[1\right],y_{3}\left[1\right],z_{3}\left[1\right]\right)=\left(100,5,0\right),$$\left(x_{A}\left[1\right],y_{A}\left[1\right],z_{A}\left[1\right]\right)=$$\left(400,100,80\right),$$\left(x_{R_{1}},y_{R_{1}},z_{R_{1}}\right)=\left(-100,50,20\right),$$\left(x_{G},y_{G},z_{G}\right)=\left(750,30,5\right),$ and $\left(x_{R_{2}},y_{R_{2}},z_{R_{2}}\right)=$$\left(900,50,20\right),$ respectively. Without loss of generality, it is considered that the vehicles are moving with a constant speed and all have an identical task requirement per timeslot. Typically, either straight-line paths or circular-orbit paths have been used for the majority of the missions of UAVs [62]. In this paper, it is considered that the ARSU flies along a pre-determined straight-line trajectory. Moreover, B=10MHz is the allocated bandwidth. Unless otherwise indicated, the values of the network parameters are listed in Table 2.

**Figure 3:**
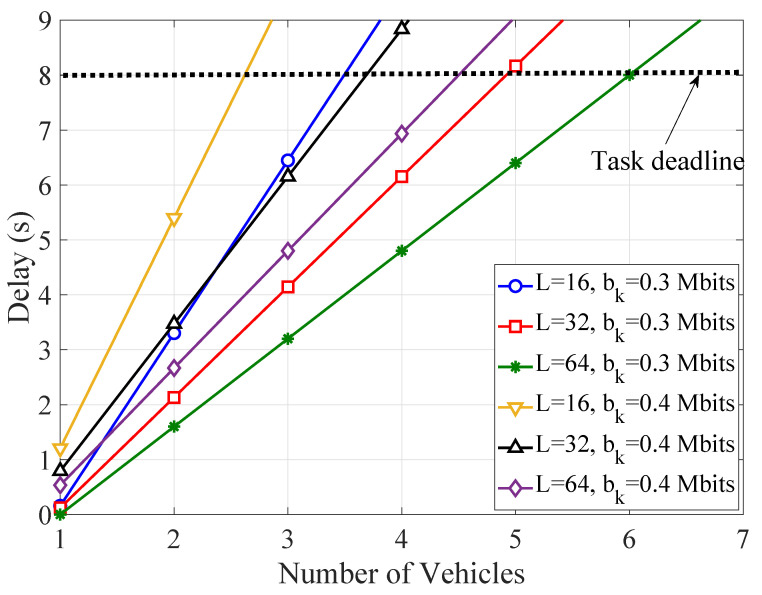
The total computation-based and communication-based delay (TCCD) as a function of the number of vehicles for different numbers of reflecting elements and task requirements.

Figure 3 depicts the TCCD as a function of the number of vehicles with a varying number of reflecting elements at RIS units and task requirements per timeslot. One observes that the delay significantly increases with the number of vehicles and the number of task bits. However, the delay decreases and more vehicles can be supported, as the number of the reflecting elements grows. For instance, RIS units with at least 64 elements are required, in order to meet the stringent latency requirements for $b_{k}\left[n\right]=0.3\;\mathrm{Mbits},$ while providing offloading services to six vehicles.

**Figure 4:**
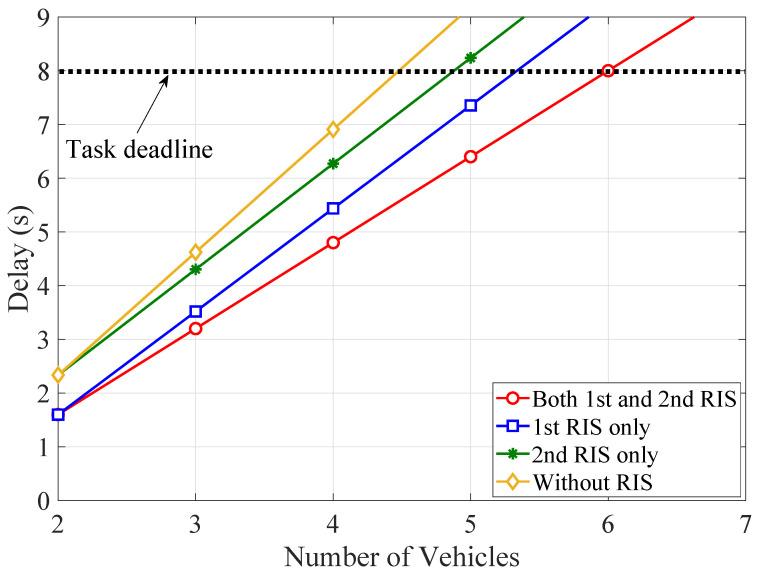
The TCCD as a function of the number of vehicles for different RIS deployment strategies.

In Figure 4, the TCCD is illustrated as a function of the number of vehicles for different configuration of RIS units and the task requirement $b_{k}\left[n\right]=0.3\;\mathrm{Mbits}$ per time slot. More specifically, the UAV-based dual-RIS, vehicles-side RIS, and GRSU-side RIS deployment strategies are investigated. In addition, a less complex UAV-based scenario is also studied, which does not include RIS units and was extensively studied in previous works (e.g., [17,18,19,20,24,25]). It is obvious that the number of supported vehicles changes by adopting a particular setup. To provide computation offloading services to more than four vehicles, while meeting the stringent latency requirements, deploying a RIS unit in the vicinity of the vehicles is at least required. Meanwhile, the dual-RIS offloading strategy supports more vehicles, when compared with the other strategies, thus highlighting the utility and feasibility of the proposed IoV architecture.

**Figure 5:**
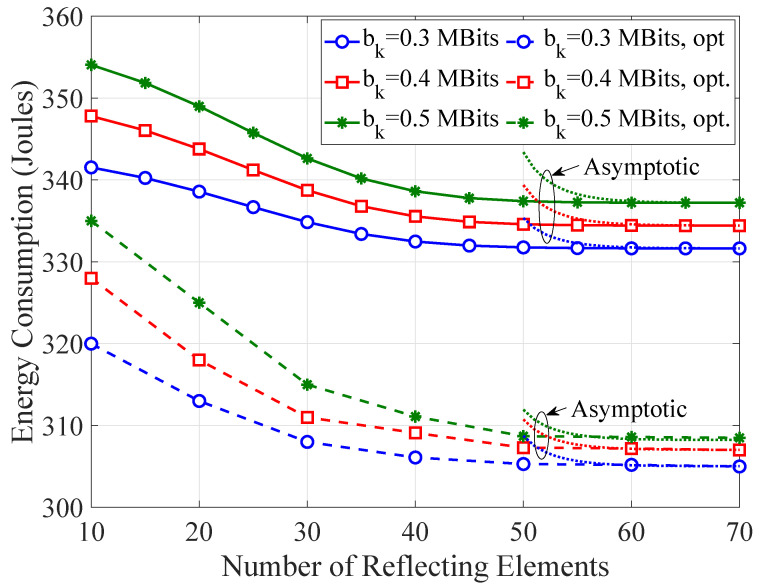
The non-optimized and optimized weighted total energy consumption (WTEC) as a function of the number of reflecting elements for varying task requirement.

Figure 5 shows the non-optimized and optimized WTEC as a function of the number of reflecting elements for varying task requirement. The asymptotic WTEC that accounts for the expressions for the asymptotic rate in (25) is also demonstrated, whereas the asymptotically optimal solutions in (35) and (36) are also verified. Clearly, the WTEC drastically decreases, as the number of the reflecting elements increases due to the lower transmission delay. As the number of these elements increases from 10 to 60, the non-optimized and optimized WTEC decreases up to 14 and 18 Joules, respectively, depending on the number of task bits. However, a reasonable number of reflecting elements should be utilized, since increasing the number of the elements beyond 60 meaninglessly changes the WTEC. Moreover, the asymptotically derived WTEC converges to the analytical WTEC with about 60 reflecting elements.

**Figure 6:**
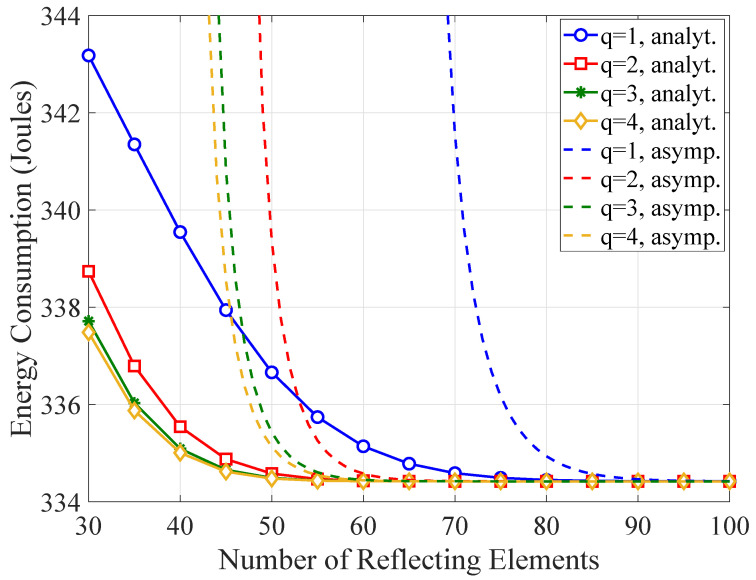
The WTEC as a function of the number of reflecting elements for a varying number of quantization bits.

The effect of the number of quantization bits on the WTEC is investigated in Figure 6. The asymptotic behavior of the WTEC is also studied. As *q* increases, more energy is consumed with a fixed number of reflecting elements. However, the WTEC is remarkably robust against phase errors for large values of reflecting elements. It can be also seen that the convergence of the asymptotic WTEC directly depends on the number of quantization bits and up to 90 reflecting elements are required for an accurate approximation of the analytical results.

**Figure 7:**
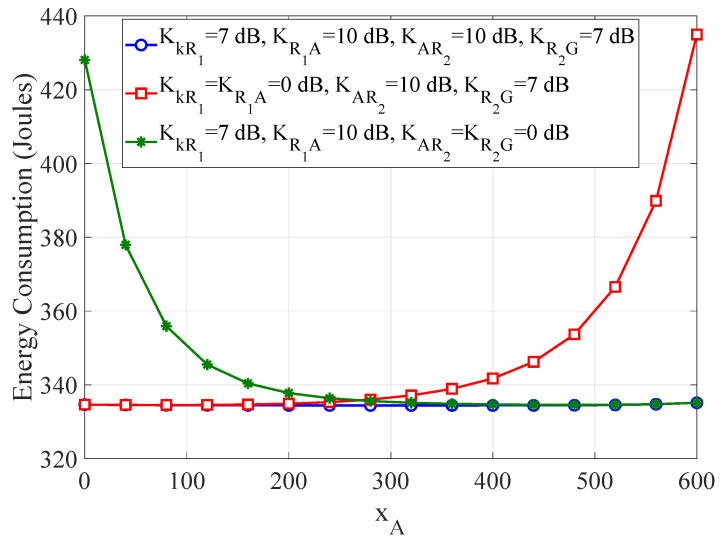
The WTEC as a function of the movement of ARSU along the *x*-axis for different values of the Rician factors $K_{kR_{1}},K_{R_{1}A},K_{AR_{2}},$ and $K_{R_{2}G}$.

Figure 7 shows the non-optimized WTEC as a function of the positioning of the ARSU along the *x*-axis for different values of the Rician factors $K_{kR_{1}},$$K_{R_{1}A},$$K_{AR_{2}},$ and $K_{R_{2}G}.$ It is obvious that the WTEC remains constant, provided that the fading is symmetric, i.e., $K_{kR_{1}}=K_{R_{2}G}=7\;\mathrm{dB}$ and $K_{R_{1}A}=K_{AR_{2}}=10\;\mathrm{dB}.$ However, the quality of the composite channel through the 1st RIS is degraded, as long as $K_{kR_{1}}=K_{R_{1}A}=0\;\mathrm{dB}.$ Then, the LoS component is weak and an ARSU position closer to the vehicles is preferable to meliorate the WTEC. On the other hand, setting $K_{AR_{2}}=K_{R_{2}G}=0\;\mathrm{dB}$ downgrades the quality of the composite channel through the 2nd RIS. Thus, moving the ARSU closer to the GRSU can compensate for this degradation. Previous results on UAV-based MEC configurations without RIS units [16] suggested a UAV position close to ground nodes to maintain low energy consumption. Nevertheless, the RIS units can enhance the communication performance of their nearby nodes and counterbalance the distance-dependent pathloss. Thus, the ARSU does not need to fly toward the vehicles and GRSU too closely, in order to obtain adequate WTEC in any fading conditions. By avoiding aimless ARSU mobility, its endurance can be significantly extended.

**Figure 8:**
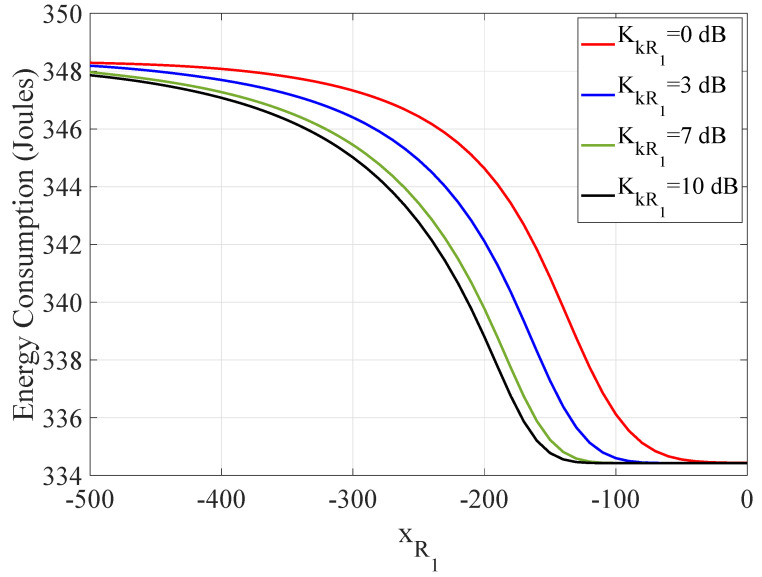
The WTEC as a function of the location of the RIS units along the x-axis for different values of the Rician factor $K_{kR_{1}}$.

In order to ascertain how the distance between the 1st RIS unit and the vehicles influences the WTEC, Figure 8 depicts the non-optimized WTEC as a function of the location of the 1st RIS unit along the *x*-axis for varying Rician factor $K_{kR_{1}}.$ One observes that the value of the WTEC is lower, when the 1st RIS unit is placed closer to the vehicles. Then, the quality of the communication link between the *k*-th vehicle and the 1st RIS unit improves mainly owing to the increase in the average SNR of the composite channel. On the other hand, the fading conditions also affect the effective rate and thus the WTEC. Hence, the vehicles should move towards the 1st RIS unit, as long as the LoS component is weak.

**Figure 9:**
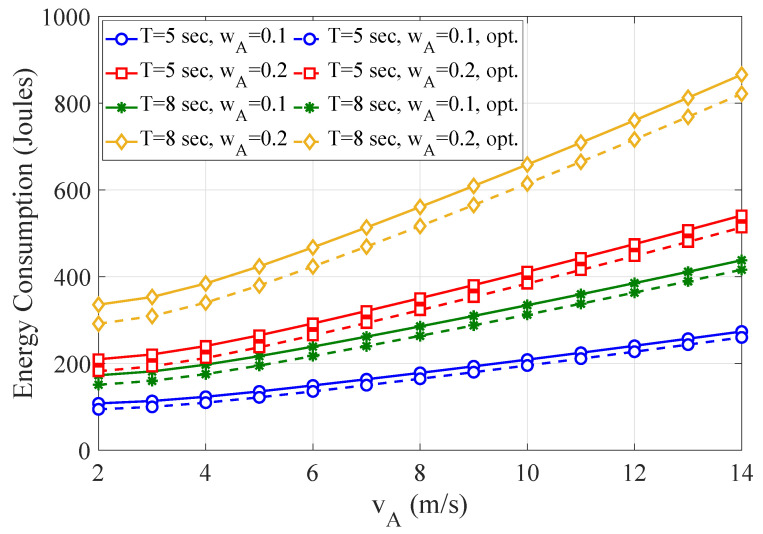
The non-optimized and optimized WTEC as a function of the velocity of ARSU for different task completion time and weight factor of energy consumption of ARSU.

Figure 9 depicts the curves of the non-optimized and optimized WTEC as a function of the velocity of ARSU for varying task completion time (flying period) and weight factor $w_{A}$ of energy consumption at ARSU. It can be observed that the consumed energy substantially increases with the velocity of ARSU owing to the growth of the propulsion energy. It is also evident that WTEC increases as *T* and $w_{A}$ step up. Moreover, the optimized scheme leads to substantially smaller values of WTEC, when compared with the non-optimized one, thus confirming the effectivity of our optimization approach.

**Figure 10:**
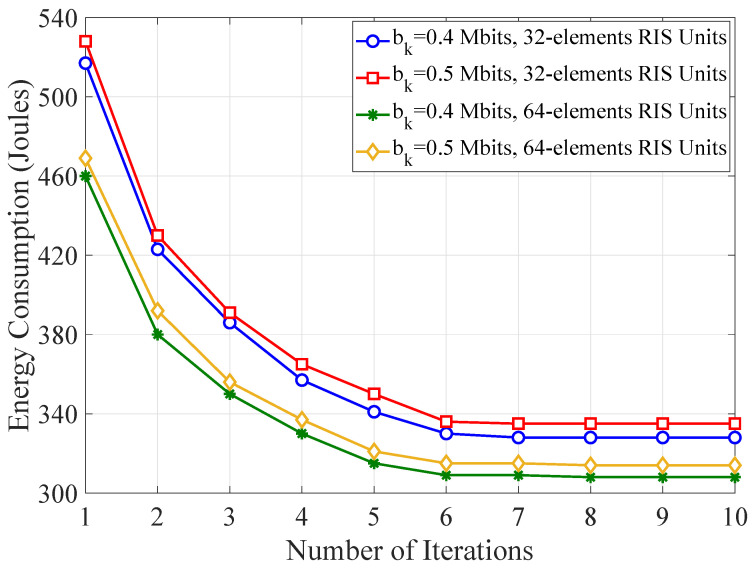
The optimized WTEC as a function of the number of iterations for varying task requirement and number of reflecting elements.

Finally, Figure 10 examines and ascertains the computational effectiveness of the proposed Algorithm 1 and shows the optimized WTEC for tolerant threshold $\epsilon =10^{-4}$ as a function of the iteration index. One observes that the optimized scheme closely converges after about seven iterations for all the combinations of task sizes and numbers of reflecting elements at RIS units.

## 6. Conclusions

This paper leveraged a UAV to provide additional computational resources and ubiquitous connectivity in future IoV networks. As RIS units constitute an emerging technology for reduced latency as well as improved energy efficiency, this paper also presented a dual-RIS network configuration and proposed a novel UAV-aided dual-RIS MEC-enabled IoV network architecture. In this direction, this paper introduced a 3-D geometrical representation of the entire network, provided an asymptotic WTEC analysis, and formulated a convex WTEC-aware optimization problem, which is subjected to several practical constraints. Based on the mathematical derivations and the convenient form of the closed-form solutions, indicative results are provided, in order to investigate the effect of the key network parameters on the non-optimized and optimized WTEC. These results underline that the number of supported vehicles is determined by the number of reflecting elements and the size of offloaded data. It is also demonstrated that the dual-RIS MEC deployment significantly outperforms other MEC deployments operating with a single RIS unit or without RIS units. Moreover, the results revealed that the impact of phase errors on the WTEC becomes less influential as the number of reflecting elements increases. Since the weight factor of ARSU and its velocity adjust the propulsion energy consumption, the results pointed out that using RIS units not only shortens the transmission delay, but also averts purposeless mobility of ARSU.

Several fertile research areas can be identified to expand this work. For instance, multiple ARSUs can be deployed to extend the network range, whereas RIS-aided WPT for flight time prolongation constitutes another interesting research direction. Furthermore, cooperative multi-RIS transmission with inter-RIS signal reflection can further improve the beamforming gain.

## Figures and Tables

**Table 1:** Synopsis of relevant research works.

References	Network Type	Key Technologies	Optimization Target
[5]	Vehicle-to-infrastructure (V2I)	Computation offloading	Lower bound of expected reliability
[6]	Vehicular network	Mobile edge computing (MEC), cloud computing	Offloading decisions
[7]	Internet of Vehicles (IoV)	MEC	Energy efficiency
[8]	Vehicular ad-hoc network (VANET)	MEC	Resource allocation
[9]	Internet of Things (IoT)	Vehicular edge computing (VEC)	Resource allocation
[10]	Vehicular network	VEC, software-defined networking (SDN)	Processing delay
[11]	Vehicle-to-vehicle (V2V) and V2I	VEC, geolocation information	Reliable data retrieval
[12]	IoV	MEC, edge intelligence	Total network delay
[16]	Cellular network	MEC, unmanned aerial vehicle (UAV)	Energy consumption
[17]	Computing system	MEC, UAV	Energy consumption
[18]	Computing system	MEC, UAV	Maximum Delay and trajectory
[19]	Computing system	MEC, UAV	Task completion time
[20]	IoT	MEC, UAV	Average latency
[21]	Computing system	MEC, UAV	Computation efficiency
[22]	Computing system	MEC, UAV, wireless power transfer (WPT)	Computation rate
[23]	IoT	Centralized and distributed MEC, UAV	Energy efficiency
[24]	IoT	MEC, UAV	Energy consumption
[25]	Social IoV (SIoV)	MEC, UAV	Resource allocation and trajectory
[26]	Vehicular network	MEC, UAV, SDN	Task execution time
[27]	Computing system	MEC, UAV, non-orthogonal multiple access (NOMA)	Bit allocation and trajectory
[28]	Computing system	MEC, UAV, stochastic offloading	Energy consumption
[29]	Vehicular network	MEC, UAV, massive multiple-input multiple-output (MIMO)	Energy consumption
[32]	Communication system	UAV, reconfigurable intelligent surface (RIS)	Achievable rate
[33]	Communication system	UAV, RIS	Sum-rate
[34]	IoT	UAV, RIS	Decoding error rate
[35]	Computing system	MEC, RIS	Latency
[36]	IoT	MEC, RIS	Sum computational bits
[37]	Computing system	MEC, RIS, NOMA	Delay
[38]	Computing system	MEC, RIS, machine learning (ML)	Learning error
This paper	IoV	MEC, UAV, RIS	Energy Consumption

**Table 2:** Definition, Notation, and Value of Network Parameters.

System and Mobility Parameters	Value
Number of vehicles: *K*	3
Weight factor for energy consumption for *k*-th vehicle (ARSU): $w_{k}\left(w_{A}\right)$	1 (0.1)
Parameters of rotary-wing UAV: $v_{tip},\;v_{0},\;d_{r},\;s,\;\rho ,\;G,\;P_{0},\;P_{1},\;P_{2}$	120, 4.3, 0.6, 0.05, 1.225, 0.503,$12\cdot 30^{3}\cdot 0.4^{3}\rho sG/8,$1.1203/2/2G, 11.46ref40
Velocity and moving direction of *k*-th vehicle in the azimuth domain, respectively: $v_{k},\gamma_{k}$	60 km/h, π/20
Velocity and moving direction of ARSU in the azimuth (elevation) domain, respectively: $v_{A},\gamma_{A,xy}\left(\gamma_{A,z}\right)$	5 m/s, 3π/2(0)
**Computation Parameters**	**Value**
Task-input data size of *k*-th vehicle per timeslot: $b_{k}$	0.4 Mbits
Task deadline (flight duration of ARSU): *T*	8 s
Timeslot length: τ	0.2 s [24]
Maximum central processing unit (CPU) frequency at ARSU: $f_{A,max}$	3 GHz [24]
Required CPU cycles per bit at ARSU: $c_{A}$	$10^{3}$ cycles/bit [24]
CPU capacitance coefficient at ARSU: $\kappa_{A}$	$10^{-27}$ [24]
**Wireless Transmission Parameters**	**Value**
Target rate: $\gamma_{t}$	1.5 bps/Hz
Max. transmit power of *k*-th vehicle and ARSU, respectively: $p_{max}^{k,off},p_{max}^{k,A,off}$	35 dBm, 35 dBm [24]
Number of reflecting elements at the 1st RIS and 2nd RIS: *L*	64
Number of quantization bits: *q*	2
Path-loss exponents: $\sigma_{kA},\sigma_{kR_{1}},\sigma_{R_{1}A},\sigma_{AG},\sigma_{AR_{2}},\sigma_{R_{2}G}$	3.5, 2.2, 2, 3.5, 2, 2.2
Channel gain at reference distance $d_{0}=1m$: $\beta_{0}$	−20 dB [32]
Variance of the additive white Gaussian noise (AWGN) at the *k*-th vehicle, ARSU, 1st RIS, ground road side unit (GRSU), and 2nd RIS: $N_{0}$	−80 dBm [32]
Nakagami-*m* fading parameter of the direct link between the *k*-th vehicle (ARSU) and ARSU (GRSU): $m_{kA}\left(m_{AG}\right)$	1 (1)
Rician factor for the link between the *k*-th vehicle and 1st RIS, 1st RIS and ARSU, ARSU and 2nd RIS, and 2nd RIS and GRSU: $K_{kR_{1}},K_{R_{1}A},K_{AR_{2}},K_{R_{2}G}$	7 dB, 10 dB, 10 dB, 7 dB
